# Forelimb preferences in quadrupedal marsupials and their implications for laterality evolution in mammals

**DOI:** 10.1186/1471-2148-13-61

**Published:** 2013-03-06

**Authors:** Andrey Giljov, Karina Karenina, Yegor Malashichev

**Affiliations:** 1 Department of Vertebrate Zoology, Saint Petersburg State University, Saint Petersburg, Russia; 2 Department of Embryology, Saint Petersburg State University, St. Petersburg, Russia

**Keywords:** Lateralization, Forelimb preference, Hand preference, Marsupial, Grey short-tailed opossum, Sugar glider, Sex effect, Postural effect, Quadruped

## Abstract

**Background:**

Acquisition of upright posture in evolution has been argued to facilitate manual laterality in primates. Owing to the high variety of postural habits marsupials can serve as a suitable model to test whether the species-typical body posture shapes forelimb preferences in non-primates or this phenomenon emerged only in the course of primate evolution. In the present study we aimed to explore manual laterality in marsupial quadrupeds and compare them with the results in the previously studied bipedal species. Forelimb preferences were assessed in captive grey short-tailed opossum (*Monodelphis domestica*) and sugar glider (*Petaurus breviceps*) in four different types of unimanual behaviour per species, which was not artificially evoked. We examined the possible effects of sex, age and task, because these factors have been reported to affect motor laterality in placental mammals.

**Results:**

In both species the direction of forelimb preferences was strongly sex-related. Male grey short-tailed opossums showed right-forelimb preference in most of the observed unimanual behaviours, while male sugar gliders displayed only a slight, not significant rightward tendency. In contrast, females in both species exhibited consistent group-level preference of the left forelimb. We failed to reveal significant differences in manual preferences between tasks of potentially differing complexity: reaching a stable food item and catching live insects, as well as between the body support and food manipulation. No influence of subjects’ age on limb preferences was found.

**Conclusions:**

The direction of sex-related differences in the manual preferences found in quadrupedal marsupials seems to be not typical for placental mammals. We suggest that the alternative way of interhemispheric connection in absence of corpus callosum may result in a fundamentally distinct mechanism of sex effect on limb preferences in marsupials compared to placentals. Our data confirm the idea that non-primate mammals differ from primates in sensitivity to task complexity. Comparison of marsupial species studied to date indicate that the vertical body orientation and the bipedalism favor the expression of individual– and population–level forelimb preferences in marsupials much like it does in primates. Our findings give the first evidence for the effect of species-typical posture on the manual laterality in non-primate mammals.

## Background

To date some level of lateralisation in forelimb use is known for a substantial number of vertebrate species from fish to mammals [1-5]. This kind of lateralization is a result of brain interhemispheric asymmetry due to differential motor activity or information processing (reviewed in [3,6,7]. Among mammals only primates historically received considerable attention and have become the subjects of detailed investigation (reviewed in [3,8,9]). At the same time, there is a growing body of evidence showing manual laterality in other mammalian groups. For instance, limb preferences at individual and/or group levels in various species-typical motor activities have been described in rodents (Mongolian gerbils [10], mice and rats [11-14]), carnivores (black bears [15], domestic cats [16-20], and dogs [21-24]), ungulates (horses [25-28], plains zebras and impalas [29], donkeys [30]), bats (Schreiber’s long-fingered bat [31]) and even in marine mammals such as pinnipeds (walruses [32]) and cetaceans (humpback whales [33], bottlenose dolphins [34], and Commerson’s dolphins [35]). Thus, some kind of phylogenetic continuity in the evolution of motor lateralization in mammals could be traced [36]. Meanwhile, the more species were studied, the more was becoming known about the specific factors shaping the expression of laterality in limb use. Animal’s posture was considered to be one of these factors.

In primates the upright posture was determined as a factor facilitating manual laterality irrespective of the bias direction: individual hand preferences significantly increased when experimental subjects had to reach a food item from a bipedal compared to a quadrupedal position (reviewed in [8,37-39]). Notably, this shift was observed not only during the comparison of the same individuals in different body postures, but also at the interspecific level. In prosimians a more vertical body orientation in a species was shown to be associated with a stronger laterality in hand use [8,40].

The species postural characteristics were proposed to influence not only the strength but also the very presence of the population level hand preference: in contrast to more upright and large-bodied species, small-bodied, quadrupedal prosimians showed no population level handedness irrespective to the subjects’ posture [39,41]. The same could be applied to some ape species. The bipedal locomotion is more typical for gorillas and gibbons than for orangutans; and indeed gorillas and gibbons are more liable to group-level handedness [42]. The latter author has hypothesised that readiness to exhibit a unilateral hand preference at the population level correlates with the degree of bipedality in a species. Furthermore, the best documented and most obvious case of behavioural laterality is pronounced handedness in humans ― the most bipedal primate [7,43]. Some authors argued that bipedalism may have facilitated species-typical right-handedness in humans [37]. This hypothesis was supported by findings in human infants, in which postural changes during early development are associated with establishing of stable handedness (reviewed in [44]). Before the age of three, infants display fluctuating patterns of manual preferences shifting together with the development of new forms of locomotion. Notably, on the stage of crawling on hands-and-knees (which in fact is a quadrupedal gait) infants exhibit no stable patterns of hand preferences, while the establishment of the latter follows closely the adoption of upright posture and bipedal locomotion.

Recently, we have explored manual laterality in a bipedal hopping marsupial: red-necked wallabies (*Macropus rufogriseus*, Diprotodontia), during their usual daily activity in zoo conditions [45]. In this work wallabies were shown to display group-level preferences to use their left forelimb in feeding from the bipedal position and to lean on the right paw in the tripedal stance. Left-forelimb bias was also traced in unimanual autogrooming. The young wallabies displayed forelimb specializations resembling that in adults: they more often used their left forelimb for pulling down the mother’s pouch and simultaneously supported the body with the right forelimb during milk suckling. In contrast, in feeding from the quadrupedal position no group-level bias was found and only a few wallabies showed individual preferences. Thus, unimanual actions performed from upright posture were more suited to reveal individual and population forelimb preferences. These results led us to a conclusion that the bipedal stance favours the expression of lateralization in wallabies [45].

Besides the subject’s and species postural characteristics, manual laterality in mammals has been shown to be influenced by such factors as sex, age, and task complexity. Sex differences in motor preferences have been described in many primates (e.g., [46-51]). Generally, bias for use the left hand is more characteristic of males, whereas a greater right-hand use has been noted for females (e.g., [8,48,50,52,53]); although a number of primate studies failed to reveal any differences in motor laterality between the sexes [54-60]. The most pronounced sex differences in manual laterality have been reported for non-primate quadrupedal mammals. In horses, domestic cats, and dogs two sexes showed oppositely directed task specializations for the forelimb use resembling primates in the tendency for females to be more right-handed and males ― more left-handed [20,22,23,26,61].

The effect of age on limb preferences also appears to be a labile category in mammals. In many species researchers have failed to reveal significant age differences in manual laterality [56,58,62-64]. However, for some primates shifts toward stronger [65-69] or weaker [46,70-72] hand preferences with age have been reported. The complexity of the manipulation task might be another factor affecting the degree of motor laterality in primates [53,70,73-77]. Fagot and Vauclair [73] suggested that in primates hand preferences at the population level most likely appear in relatively complex tasks requiring postural, perceptual or cognitive demands, such as bimanual manipulation or catching live prey. For instance, in squirrel monkeys group-level preference of the left hand was expressed in catching a fish, but was absent in reaching of stable food item, i.e., simple reaching [78].

Despite that great number of mammalian species studied to date in terms of behavioural lateralization in general and in particular in the aspect of manual laterality, on closer inspection, the set of studied taxa still has a number of white spaces. One of such an underrepresented group is the marsupial mammals. To the best of our knowledge, studies of marsupial laterality existing to date only report sensory lateralization in captive stripe-faced dunnarts [79] and hairy-nosed wombats [80], individual forelimb preferences in captive brush-tailed possums [81], and grey short-tailed opossums [82], as well as population-level manual laterality in captive red-necked wallabies [45]. Nonetheless, data on asymmetrical limb use in marsupials would lead to gain a broader picture of the motor laterality evolution and its possible adaptive value. In addition, the comparison of lateralized limb use between the placentals and marsupials is important because these groups developed in parallel during evolution [83,84] and share largely similar ecological adaptations and lifestyles.

Marsupials appear to be good candidates for investigating the effect of posture on the manifestation of forelimb preferences. Overall, it is considered that the species-typical posture interacts with the expression of manual preferences in primates. However, very little is known about whether this point is applicable to non-primates. Konerding et al. [85] showed that in domestic cats forelimb preferences are not affected by task’s postural demands. No differences in the direction or the strength of cats’ lateral biases were revealed between two variants of unimanual task ― food grasping from stable (sitting or standing) vs. unstable body posture (vertical clinging). To our knowledge, only this study together with the one on wallabies [45] aimed to investigate postural effect on laterality in non-primate mammals. It is, thus, clear that further investigation is needed to understand whether the body posture could shape manual laterality in non-primates or this phenomenon emerged only in the course of primate evolution. Because of the high diversity of postural habits and gaits varying from obligatory quadrupedal to entirely bipedal locomotion [86,87], marsupials are a suitable model to gain insight into this issue. In red-necked wallabies the bipedal posture was found to increase the laterality [45], but whether the postural effect across species takes place in marsupials is not known. Since we have previously investigated lateral forelimb biases in a bipedal hopping marsupial, i.e., the species with bipedal locomotion as the preferred gait, we now aimed to study limb preferences in marsupial quadrupeds and compare them with the results in the bipedal species. Here we examine the forelimb preferences at the individual and population levels in two marsupial species, whose typical mode of locomotion is walking and climbing on all four limbs: grey short-tailed opossums*, Monodelphis domestica* (Didelphimorphia, Didelphidae) and sugar gliders, *Petaurus breviceps* (Diprotodontia, Petauridae). Basing on the primate data, demonstrating that quadrupedal locomotion tends to hinder the expression of handedness [8,37,42,44], we hypothesized that quadrupedal marsupials should be less lateralized at individual and population level than the bipedal one.

The principal aim of the present study was to explore first the influence of the main factors affecting the motor preferences in placental mammals (such as species-typical posture, sex, age and task complexity) on manual laterality in marsupials. Consistency between the effects of these factors in marsupials and placentals as well as the implication of marsupial data for the current theories of manual laterality is then discussed.

All animals used in the present study were housed in the zoo. Forelimb preferences in both species were investigated in four types of unimanual behaviour: feeding on non-living food, feeding on live insects, supporting the body in the tripedal stance, and nest-material collecting. Unimanual behaviours were not artificially evoked. The animals were video recorded during their usual activity in the dark phase of day-night cycle using the cameras with infrared lighting. Video recording was conducted outside the cages to minimize possible disturbance.

## Results

### Feeding on non-living food

After we reduced the data for each individual to the smallest value obtained in the group (see Methods), there remained: 45 unimanual acts per individual in *M. domestica* and 28 unimanual acts per individual in *P. breviceps* for feeding on non-living food.

**Figure 1:**
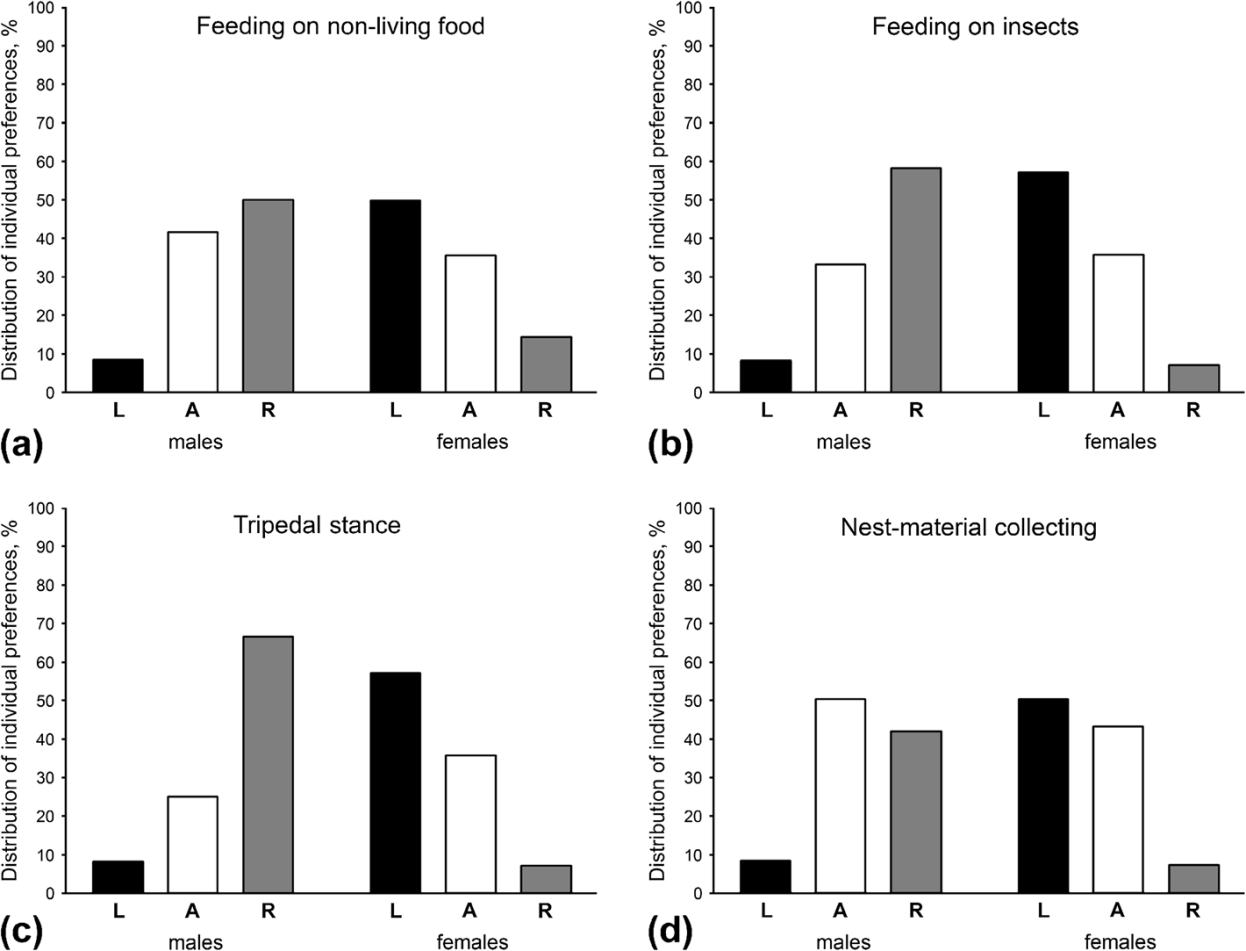
**Percentage distribution of left-handed (L), right-handed (R) and ambipreferent (A) individuals in grey short-tailed opossums. **The direction of manual lateralization in all types of behaviour studied (**a,b,c,d**) was found to be significantly influenced by the animals’ sex, therefore distribution is given separately for males, N = 12, and females, N = 14. Reversed distributions of limb preferences among subjects in two sexes could be traced: right-forelimb preference is more characteristic of males, while left-forelimb preferences are more typical for females.

**Figure 2:**
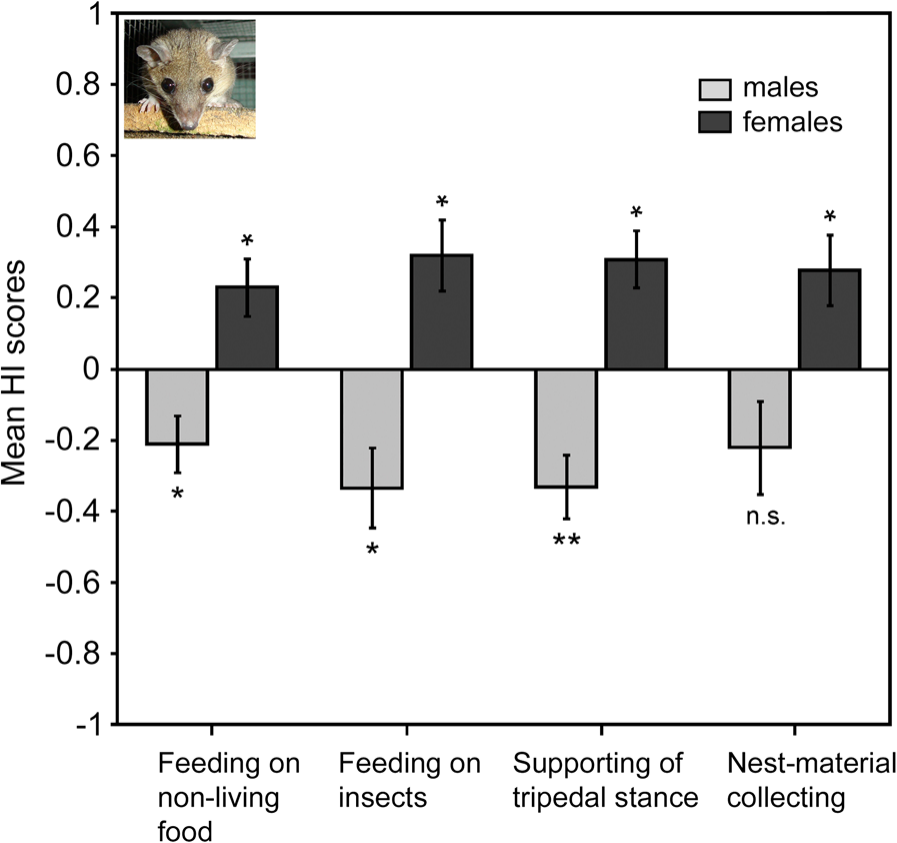
**Direction of limb preferences in grey short-tailed opossums. **Direction is given separately for male, N = 12, and female, N = 14 groups. Mean HI scores ± SE for four types of unimanual behaviour (positive values indicate left lateral bias, negative values indicate right lateral bias).*P < 0.05;**P < 0.005; n.s.: non-significant. Significant group-level preference of the left forelimb was found in females in all types of unimanual behaviour studied. Males exhibit no group preference in nest-material collecting and right-forelimb preference in the rest of behaviours.

In *M. domestica* the distribution of individual forelimb preferences did not differ from chance (χ^2^_2_ = 1.39, *P* = 0.500) with eight left-forelimb preferent opossums, eight ― right-forelimb preferent, and 10 ambipreferent subjects (Table 1). However, males and females displayed somewhat reversed distributions of limb preferences among subjects with slightly more left-forelimb preferent females and more males showing right-forelimb preferences (Figure 1a). The direction, but not the strength of manual lateralization was significantly influenced by the animals’ sex (Mann–Whitney *U* test: *U* = 28.00, *P* = 0.004 for direction, and *U* = 73.50, *P* = 0.605 for strength). Significant group-level right-forelimb preference was found for *M. domestica* males (mean HI = −0.21 ± 0.08; one-sample Wilcoxon Signed-rank test: *Z* = −53.00, *P* = 0.041, *N* = 12; Figure 2), while in females we found significant group-level preference of the left forelimb (mean HI = 0.23 ± 0.08; one-sample Wilcoxon Signed-rank test: *Z* = 66.00, *P* = 0.041, *N* = 14; Figure 2). The analysis failed to reveal any significant correlation between the age of males and both the direction (Spearman rank-order correlation: *r*_s_ = −0.12, *P* = 0.712) and strength of forelimb preferences (*r*_s_ = −0.02, *P* = 0.938). In females age also did not correlate with either the direction (Spearman rank-order correlation: *r*_s_ = −0.37, *P* = 0.190) or strength of preferences (*r*_s_ = −0.20, *P* = 0.491).

**Table 1:** Individual forelimb preferences in grey short-tailed opossum

**Subject**	**Sex**	**Age**	**Feeding on**						**Supporting of tripedal stance**			**Nest-material collecting**		
			**Non-living food**			**Live insects**								
			**HI**	***z***	**Pref**	**HI**	***z***	**Pref**	**HI**	***z***	**Pref**	**HI**	***z***	**Pref**
1	M	34	0.20	1.19	A	−0.03	0.00	A	−0.15	−0.18	A	−0.71	−3.94	R
2	M	21	−0.56	−3.72	R	−0.68	−3.72	R	−0.65	−3.95	R	−0.65	−3.72	R
3	M	2	0.02	0.00	A	0.48	−2.55	R	0.20	1.11	A	0.24	1.20	A
4	M	14	0.38	2.41	L	0.48	2.55	L	0.35	2.07	L	0.65	3.72	L
5	M	17	−0.51	−3.35	R	−0.35	−1.81	A	−0.45	−2.73	R	−0.53	−2.98	R
6	M	6	−0.33	−2.10	R	0.48	−2.55	R	−0.55	−3.39	R	−0.29	−1.55	A
7	M	5	−0.29	−1.80	A	−0.55	−2.93	R	−0.60	−3.72	R	−0.35	−1.90	A
8	M	4	−0.29	−1.80	A	−0.10	−0.36	A	−0.30	−1.75	A	0.12	0.51	A
9	M	6	−0.38	−2.41	R	−0.61	−3.32	R	−0.45	−2.73	R	−0.41	−2.25	R
10	M	2	−0.33	−2.10	R	−0.42	−2.18	R	−0.40	−2.40	R	0.24	1.20	A
11	M	3	−0.07	−0.30	A	0.03	0.00	A	−0.40	−2.40	R	−0.12	−0.51	A
12	M	3	−0.42	−2.72	R	−0.68	−3.72	R	−0.50	−3.06	R	−0.76	−4.29	R
13	F	6	0.16	0.89	A	−0.29	−1.44	A	−0.55	−3.39	R	0.00	0.00	A
14	F	21	0.29	1.80	A	0.23	1.08	A	0.10	0.47	A	−0.12	−0.51	A
15	F	21	0.11	0.60	A	0.10	0.36	A	0.40	2.40	L	0.59	3.16	L
16	F	17	−0.38	−2.41	R	0.61	3.32	L	0.70	4.27	L	0.29	1.55	A
17	F	14	−0.47	−3.02	R	−0.55	−2.93	R	0.05	0.16	A	−0.24	−1.20	A
18	F	14	0.33	2.10	L	0.61	3.32	L	0.35	2.07	L	0.47	2.61	L
19	F	7	0.24	1.49	A	0.35	1.81	A	0.25	1.43	A	0.06	0.17	A
20	F	7	0.33	2.10	L	0.42	2.18	L	0.50	3.06	L	0.41	2.25	L
21	F	7	0.24	1.49	A	0.48	2.55	L	0.20	1.11	A	−0.47	−2.61	R
22	F	39	0.60	3.88	L	0.74	3.95	L	0.60	3.72	L	0.47	2.61	L
23	F	5	0.38	2.41	L	0.35	1.81	A	0.35	2.07	L	0.35	1.90	A
24	F	5	0.51	3.35	L	0.42	2.18	L	0.45	2.73	L	0.82	4.63	L
25	F	2	0.42	2.72	L	0.42	2.18	L	0.30	1.75	A	0.59	3.16	L
26	F	2	0.51	3.35	L	0.55	2.93	L	0.60	3.72	L	0.71	3.94	L

(HI: handedness index; *z*: z score, positive values indicate leftward bias, negative values indicate rightward bias; Pref: forelimb preference, L: left forelimb; R: right forelimb; A: ambipreferent. Age is given in months).

**Figure 3:**
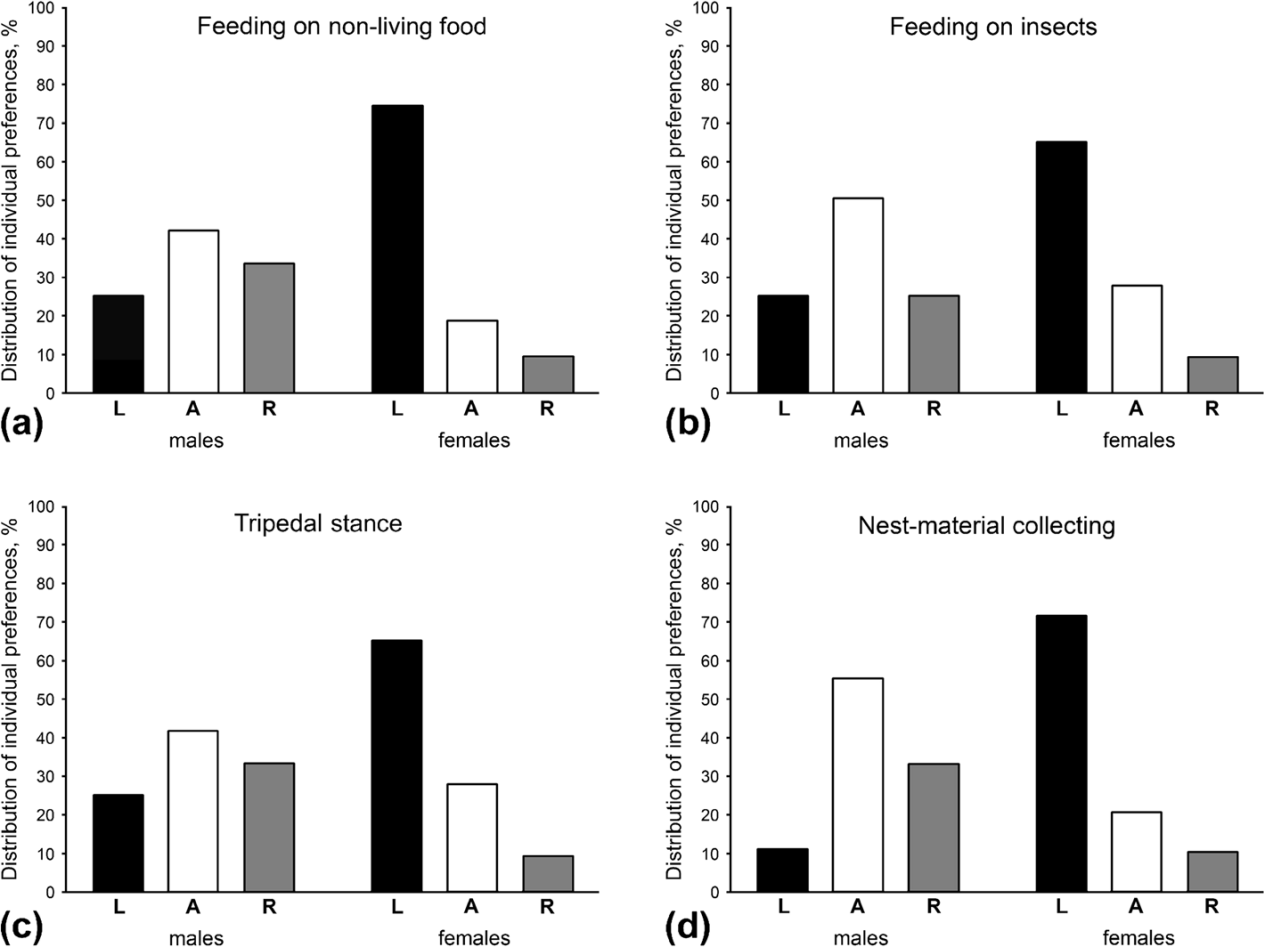
**Percentage distribution of left-handed (L), right-handed (R) and ambipreferent (A) individuals in sugar gliders. **The direction of manual lateralization in all types of behaviour studied was found to be significantly influenced by the animals’ sex, therefore distribution is given separately for two sexes (feeding **(a,b) **and tripedal stance **(c)**: 12 males, 11 females; nest-material collecting **(d)**: 9 males, 10 females). Distribution of subject’s preferences tended to be more symmetrical in males, than in females, among whom there were more left-handers.

**Figure 4:**
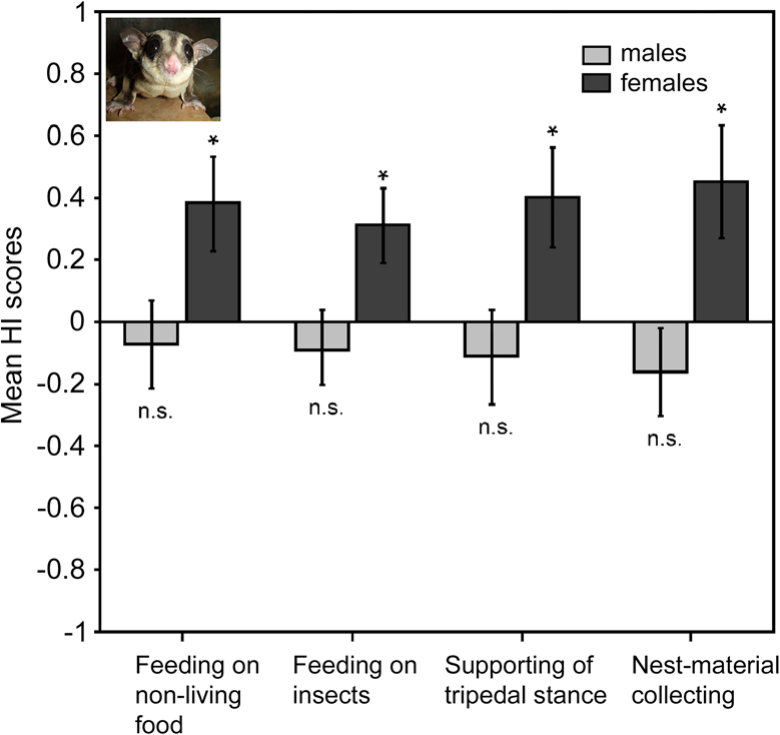
**Direction of limb preferences in sugar gliders. **Direction is given separately for in male and female groups (feeding and tripedal stance: 12 males, 11 females; nest-material collecting: 9 males, 10 females). Mean HI scores ± SE for four types of unimanual behaviour (positive values indicate left lateral bias, negative values indicate right lateral bias). *P < 0.05; n.s.: non-significant. In all behaviours females showed group-level preference for use of the left forelimb, while males had no across group consistent preference.

In *P. breviceps* the distribution of individual forelimb preferences for feeding on non-living food differed significantly from chance (χ^2^_2_ = 6.65, *P* = 0.036), with 11 left-forelimb preferent sugar gliders, five right-forelimb preferent subjects, and seven subjects which had no significant preference (Table 2). However, the distributions of subject’s preferences were more symmetrical in males, than in females, among whom there were more left-handers (Figure 3а). As in opossums, in sugar gliders we found significant influence of the animals’ sex on the direction, but not the strength of forelimb preferences, though, not so pronounced (Mann–Whitney *U* test: *U* = 33.00, *P* = 0.045 for direction and *U* = 47.50, *P* = 0.266 for strength). In males no significant group-level preference was found (mean HI = −0.07 ± 0.14; one-sample Wilcoxon Signed-rank test: *Z* = −12.00, *N* = 12, *P* = 0.666; Figure 4). In contrast to males, however, group-level preference of the left forelimb was found in females (mean HI = 0.38 ± 0.15; one-sample Wilcoxon Signed-rank test: *Z* = 53.00, *N* = 11, *P* = 0.008; Figure 4). Like in opossums, in male sugar gliders the age of individuals did not significantly influence either the direction (Spearman rank-order correlation: *r*_s_ = −0.06, *P* = 0.845) or strength of forelimb preferences (*r*_s_ = 0.11, *P* = 0.726). In females also no significant correlation was found between the age of individuals and both the direction (Spearman rank-order correlation: *r*_s_ = −0.38, *P* = 0.243) and strength of forelimb preferences (*r*_s_ = −0.28, *P* = 0.397).

**Table 2:** Individual forelimb preferences in sugar glider

**Subject**	**Sex**	**Age**	**Feeding on**						**Supporting of tripedal stance**			**Nest-material collecting**		
			**Non-living food**			**Live insects**								
			**HI**	***z***	**Pref**	**HI**	***z***	**Pref**	**HI**	***z***	**Pref**	**HI**	***z***	**Pref**
1	M	42	−0.50	−2.50	R	−0.22	−1.17	A	−0.49	−3.26	R	−0.36	−1.61	A
2	M	25	−0.21	−0.94	A	−0.17	−0.83	A	−0.62	−4.08	R	−0.60	−2.87	R
3	M	27	−0.36	−1.71	A	0.11	0.50	A	−0.15	−0.87	A	−0.20	−0.80	A
4	M	15	−0.71	−3.54	R	−0.50	−2.89	R	−0.23	−1.46	A	–	–	–
5	M	16	0.43	1.80	L	0.39	2.19	L	0.79	5.25	L	0.76	3.60	L
6	M	13	−0.14	−0.57	A	−0.22	−1.17	A	−0.06	−0.29	A	−0.52	−2.44	R
7	M	13	0.64	3.32	L	0.50	2.89	L	0.28	1.76	A	0.12	0.40	A
8	M	12	−0.50	−2.24	R	−0.44	−2.28	R	−0.66	−4.38	R	–	–	–
9	M	6	−0.57	−2.90	R	−0.89	−5.17	R	−0.87	−5.83	R	−0.44	−2.02	R
10	M	10	0.14	0.57	A	−0.28	−1.50	A	−0.23	−1.46	A	−0.28	−1.20	A
11	M	15	0.79	3.97	L	0.56	3.24	L	0.40	2.66	L	–	–	–
12	M	6	0.21	0.94	A	0.11	0.50	A	0.57	3.79	L	0.04	0.00	A
13	F	48	0.71	3.54	L	0.67	3.83	L	0.87	5.83	L	0.84	4.00	L
14	F	46	0.43	1.80	L	0.39	2.19	L	−0.06	−0.29	A	0.60	2.87	L
15	F	45	−0.07	−0.19	A	−0.17	−0.83	A	−0.15	−0.87	A	−0.68	−3.32	R
16	F	25	−0.86	−4.35	R	−0.50	−2.89	R	−0.75	−4.96	R	−0.28	−1.20	A
17	F	26	0.21	0.94	A	0.56	3.24	L	1.00	6.71	L	0.76	3.60	L
18	F	24	0.50	2.50	L	0.61	3.62	L	0.45	2.96	L	0.52	2.44	L
19	F	17	0.79	3.97	L	0.72	4.17	L	0.70	4.38	L	0.92	4.40	L
20	F	13	0.64	3.32	L	0.61	3.62	L	0.75	4.96	L	0.68	3.32	L
21	F	8	0.79	3.97	L	−0.11	−0.50	A	0.28	1.76	A	0.12	0.40	A
22	F	10	0.43	1.80	L	0.22	1.17	A	0.40	2.66	L	–	–	–
23	F	12	0.57	2.90	L	0.44	2.28	L	0.91	6.13	L	1.00	4.80	L

(HI: handedness index; z: z score, positive values indicate leftward bias, negative values indicate rightward bias; Pref: forelimb preference, L: left forelimb; R: right forelimb; A: ambipreferent. Age is given in months).

### Feeding on live insects

After reduction of the data for each individual to the smallest value obtained in the group, we obtained 31 acts per individual in *M. domestica* and 36 acts per individual in *P. breviceps*.

The distribution of forelimb preferences in *M. domestica* did not differ significantly from chance (χ^2^_2_ = 0.08, *P* = 0.962): nine opossums showed the left-forelimb preference, eight opossums ― the right-forelimb preference, and nine individuals were classified as ambipreferent (Table 1). Similarly to feeding on non-living food, when divided by sex, more females were distributed among left-forelimb preferent subjects and slightly more males showed preference of the right forelimb (Figure 1b). The sex of animals significantly influenced the direction (Mann–Whitney *U* test: *U* = 20.00, *P* = 0.001), but not the strength of manual lateralization (*U* = 47.50, *P* = 0.266). Significant group–level right-forelimb preference was revealed in males (mean HI = −0.32 ± 0.10; one-sample Wilcoxon Signed-rank test: *Z* = −61.00, *P* = 0.018, *N* = 12; Figure 2), while in females significant group–level preference of the left forelimb was found (mean HI = 0.32 ± 0.10; one-sample Wilcoxon Signed-rank test: *Z* = 78.00, *P* = 0.016, *N* = 14; Figure 2). Age was not the factor associated with either the direction (Spearman rank-order correlation: *r*_s_ = 0.09, *P* = 0.778) or strength (*r*_s_ = 0.24, *P* = 0.459) of manual laterality in males, as well as in females (*r*_s_ = 0.05, *P* = 0.852 for direction and *r*_s_ = 0.12, *P* = 0.682 for strength).

In case of *P. breviceps*, ten subjects showed left-forelimb preference, four ― right-forelimb preference and nine had no significant preference of one of the forelimbs in feeding on live insects (Table 2). This distribution did not differ significantly from chance (χ^2^_2_ = 4.22, *P* = 0.121). Sex differences in distribution of forelimb preferences between sexes could also be traced, with right-sided preferences more expressed in males, and left-sided preferences ― in females (Figure 3b). The Mann–Whitney *U* tests revealed that the sex of animals significantly influenced the direction (*U* = 29.00, *P* = 0.024), but not the strength of manual lateralization (*U* = 47.00, *P* = 0.253). No significant group–level preference was found in males (mean HI = −0.09 ± 0.13; one-sample Wilcoxon Signed-rank test: *Z* = −17.00, *N* = 12, *P* = 0.530; Figure 4). In females, in contrast, the group–level preference of the left forelimb was revealed (mean HI = 0.32 ± 0.12; one-sample Wilcoxon Signed-rank test: *Z* = 49.00, *N* = 11, *P* = 0.014; Figure 4). Age did not correlate with either the direction (Spearman rank–order correlation: *r*_s_ = 0.33, *P* = 0.297) or strength (*r*_s_ = −0.30, *P* = 0.341) of manual laterality in males, as well as in females (*r*_s_ = 0.13, *P* = 0.706 for direction and *r*_s_ = 0.29, *P* = 0.387 for strength).

### Supporting the body in the tripedal stance

For the unimanual forelimb use in tripedal stance, after data cutting, we obtained 40 acts of tripedal positioning per individual in *M. domestica* and 47 unimanual acts per individual in *P. breviceps*.

In *M. domestica* on the basis of individual binomial *z* scores nine opossums were classified as left-forelimb preferent, nine opossums ― as right-forelimb preferent and eight ― did not show significant preference (Table 1). This distribution of forelimb preferences did not differ from chance (χ^2^_2_ = 3.85, *P* = 0.146). Sex differences in distribution of forelimb preferences between sexes could be also traced, with more left-forelimb preferent subjects among females and right-forelimb preferent subjects among males (Figure 1c). The direction, but not the strength of manual lateralization was significantly influenced by the animals’ sex (Mann–Whitney *U* test: *U* = 18.00, *P* < 0.001 for direction, and *U* = 76.00, *P* = 0.699 for strength). Again, significant group-level right-forelimb preference was revealed in males (mean HI = −0.33 ± 0.09; one-sample Wilcoxon Signed-rank test: *Z* = −66.00, *P* = 0.011, *N* = 12; Figure 2), while in females significant group-level preference of the left forelimb was found (mean HI = 0.31 ± 0.08; one-sample Wilcoxon Signed-rank test: *Z* = 83.00, *P* = 0.010, *N* = 14; Figure 2). The analysis failed to reveal any significant correlation between the age of subjects and both the direction (Spearman rank-order correlation: *r*_s_ = −0.17, *P* = 0.594) and strength (*r*_s_ = −0.08, *P* = 0.815) of manual laterality in males, as well as in females (*r*_s_ = 0.07, *P* = 0.814 for direction and *r*_s_ = −0.03, *P* = 0.906 for strength).

For *P. breviceps* ten individuals were classified as left-forelimb preferent, five ― as right-forelimb preferent, and eight subjects were classified as ambipreferent (Table 2). This distribution of forelimb preferences did not differ more than would be expected by chance (χ^2^_2_ = 4.30, *P* = 0.116). Sex differences in distribution of individual preferences, similar to those observed in feeding, could be traced, with slightly more left-handers among females (Figure 3c). The sex of subjects significantly influenced the direction (Mann–Whitney *U* test: *U* = 31.00, *P* = 0.034), but not the strength of manual laterality (*U* = 49.00, *P* = 0.309). No forelimb preference at the group–level was revealed in males (mean HI = −0.11 ± 0.15; one-sample Wilcoxon Signed-rank test: *Z* = −18.00, *P* = 0.505, *N* = 12; Figure 4), whereas females displayed significant group–level preference of the left forelimb (mean HI = 0.40 ± 0.16; one-sample Wilcoxon Signed-rank test: *Z* = 49.00, *P* = 0.010, *N* = 11; Figure 4). Age was not associated with either the direction (Spearman rank–order correlation: *r*_s_ = 0.06, *P* = 0.861) or strength (*r*_s_ = −0.19, *P* = 0.549) of manual lateralization in males, as well as in females (*r*_s_ = −0.06, *P* = 0.854 for direction and *r*_s_ = 0.02, *P* = 0.946 for strength).

### Nest-material collecting

In forelimb use during nest-material collecting, after we reduced individual data to the smallest value obtained in the group, we had received 34 acts per individual in *M. domestica* and 25 unimanual acts per individual in *P. breviceps*.

In *M. domestica* the distribution of individual preferences did not differ more than would be expected by chance (χ^2^_2_ = 2.15, *P* = 0.340): eight opossums showed preference to use the left forelimb, six ― the right forelimb, and 12 had no significant preference (Table 1). However, some sex differences in distribution of subjects’ forelimb preferences could also be traced (Figure 1d). A significant influence of the individuals’ sex on the direction, but not the strength of forelimb preferences was found (Mann–Whitney *U* test: *U* =32.50, *P* = 0.009 for direction and *U* = 80.00, *P* = 0.857 for strength). In females the group–level preference of the left forelimb was found (mean HI = 0.38 ± 0.15; one-sample Wilcoxon Signed-rank test: *Z* = 65.00, *N* = 14, *P* = 0.025; Figure 2). In males, however, forelimb preference in nest-material collecting at the level of group did not reach significance, although it was slightly skewed to the right (mean HI = −0.22 ± 0.13; one-sample Wilcoxon Signed-rank test: *Z* = −42.00, *N* = 12, *P* = 0.107; Figure 2).

Age did not correlate with either the direction (Spearman rank–order correlation: *r*_s_ = −0.48, *P* = 0.116) or strength (*r*_s_ = 0.40, *P* = 0.198) of manual laterality in males, as well as in females (*r*_s_ = −0.31, *P* = 0.281 for direction and *r*_s_ = −0.29, *P* = 0.323 for strength).

In case of *P. breviceps* eight subjects showed significant preference of the left forelimb, four — of the right forelimb, and seven had no significant preference (Table 2). This distribution of forelimb preferences did not differ from chance (χ^2^_2_ = 1.37, *P* = 0.505). Again, males and females displayed slightly different distributions of preferences across subjects (Figure 3d).

The Mann–Whitney *U* tests revealed that sex of animals significantly affected both the direction (*U* = 18.50, *P* = 0.034), and strength of manual lateralization (*U* = 19.50, *P* = 0.041), with females displayed stronger manual laterality than males. No significant group-level preference was found in males (mean HI = −0.16 ± 0.14; one-sample Wilcoxon Signed-rank test: *Z* = −21.00, *N* = 9, *P* = 0.250; Figure 4). In females analysis revealed significant preference of the right forelimb at the group level (mean HI = 0.45 ± 0.18; one-sample Wilcoxon Signed-rank test: *Z* = 36.00, *N* = 10, *P* = 0.038; Figure 4). Age did not correlate with either the direction (Spearman rank–order correlation: *r*_s_ = −0.08, *P* = 0.830) or strength (*r*_s_ = 0.29, *P* = 0.456) of manual laterality in males, as well as in females (*r*_s_ = 0.11, *P* = 0.742 for direction and *r*_s_ = 0.09, *P* = 0.816 for strength).

### Comparison across types of behaviour

In the *M. domestica* the type of behaviour had no significant effect on the direction of forelimb preferences either in males (Friedman’s test: χ^2^_3_ = 6.90, *P* = 0.075), or in females (Friedman’s test: χ^2^_3_ = 0.28, *P* = 0.964). The same was true for *P. breviceps* males (Friedman’s test: χ^2^_3_ = 1.93, *P* = 0.586) and females (Friedman’s test: χ^2^_3_ = 4.44, *P* = 0.218).

The strength of manual preferences also was not associated with the type of behaviour either in *M. domestica* males (Friedman’s test: χ^2^_3_ =4.40, *P* = 0.221) or females (Friedman’s test: χ^2^_3_ =2.14, *P* = 0.545), as well as in *P. breviceps* males (Friedman’s test: χ^2^_3_ =1.40, *P* = 0.706) and females (Friedman’s test: χ^2^_3_ = 4.44, *P* = 0.218).

Comparison between two types of feeding (on non-living food and on live insects) did not reveal any difference either in the direction (Wilcoxon matched-pairs signed rank test: *Z* = 2.00, *P* = 0.970) or strength (*Z* = −34.00, *P* = 0.204) of forelimb preferences in *M. domestica* males, as well as in females (*Z* = −29.00, *P* = 0.391 for direction and *Z* = −31.00, *P* = 0.358 for strength). In *P. breviceps* no significant difference in manual laterality between two types of feeding was found either in the direction (Wilcoxon matched pairs test for feeding: *Z* = 12.00, *P* = 0.677) or strength (*Z* = 34.00, *P* = 0.204) of forelimb preferences of males, as well as of females (*Z* = 16.00, *P* = 0.520 for direction and *Z* = 26.00, *P* = 0.278 for strength).

Significant positive correlations were found between the HI scores for all four types of behaviour in both opossums (Spearman rank–order correlation: feeding on non-living food vs. feeding on live insects: *r*_*s*_ = 0.78, *P* < 0.001; feeding on non-living food vs. tripedal stance: *r*_*s*_ = 0.69, *P* < 0.001; feeding on non-living food vs. nest-material collecting: *r*_*s*_ = 0.72, *P* < 0.001; feeding on live insects vs. tripedal stance: *r*_*s*_ = 0.86, *P* < 0.001; feeding on live insects vs. nest-material collecting: *r*_*s*_ = 0.70, *P* < 0.001; tripedal stance vs. nest-material collecting: *r*_*s*_ = 0.79, *P* < 0.001) and sugar gliders (Spearman rank-order correlation: feeding on non-living food vs. feeding on live insects: *r*_*s*_ = 0.80, *P* < 0.001; feeding on non-living food vs. tripedal stance: *r*_*s*_ = 0.75, *P* < 0.001; feeding on non-living food vs. nest-material collecting: *r*_*s*_ = 0.75, *P* < 0.001; feeding on live insects vs. tripedal stance: *r*_*s*_ = 0.83, *P* < 0.001; feeding on live insects vs. nest-material collecting: *r*_*s*_ = 0.83, *P* < 0.001; tripedal stance vs. nest-material collecting: *r*_*s*_ = 0.86, *P* < 0.001).

### Species comparison

A comparison of the overall strength of forelimb preferences between the two species found the sugar gliders (mean ABS–HI ± SEM = 0.49 ± 0.05) to be more strongly lateralized in feeding on non-living food than the grey short-tailed opossums (mean ABS–HI ± SEM = 0.34 ± 0.03; Mann–Whitney *U* test: *U* = 180.00, *P* = 0.018). However no differences in strength of manual preferences were revealed between the species in the other three types of behaviour (Mann–Whitney *U* test: feeding on live insects: *U* = 286.00, *P* = 0.802; supporting of tripedal position: *U* = 234.00, *P* = 0.196; nest-material collecting: *U* = 192.00, *P* = 0.210.)

When separated by sex, no difference between male *M. domestica* and male *P. breviceps* was found in either the direction (Mann–Whitney *U* test: feeding on non-living food: *U* = 63.00, *P* = 0.623; feeding on live insects: *U* = 45.00, *P* = 0.126; supporting of tripedal position: *U* = 54.00, *P* = 0.312; nest-material collecting: *U* = 49.00, *P* = 0.749) or strength of forelimb preferences (Mann–Whitney *U* test: feeding on non-living food: *U* = 49.00, *P* = 0.193; feeding on live insects: *U* = 64.00, *P* = 0.664; supporting of tripedal position: *U* = 67.00, *P* = 0.795; nest-material collecting: *U* = 47.00, *P* = 0.644). The direction of manual laterality did not differ between female *M. domestica* and female *P. breviceps* (Mann–Whitney *U* test: feeding on non-living food: *U* = 46.00, *P* = 0.095; feeding on live insects: *U* = 72.00, *P* = 0.805; supporting of tripedal position: *U* = 60.00, *P* = 0.366; nest-material collecting: *U* = 45.00, *P* = 0.151). However, forelimb preferences of female sugar gliders were stronger when compared to opossums in feeding on non-living food (Mann–Whitney *U* test: *U* = 38.00, *P* = 0.035) and in nest-material collecting (*U* = 32.00, *P* = 0.028). Strength of manual lateralization in other two types of behaviour did not differ between females of the two species (Mann–Whitney *U* test: feeding on live insects: *U* = 70.00, *P* = 0.722; supporting of tripedal position: *U* = 48.00, *P* = 0.119).

## Discussion

The present study revealed that studied samples of two captive quadrupedal marsupials, grey short-tailed opossums and sugar gliders, are comprised of ambipreferent, left-forelimb preferent, and right-forelimb preferent individuals in approximately equal numbers in most of the unimanual behaviours studied. In only one type of behaviour, feeding on non-living food, the distribution of individual forelimb preferences in sugar gliders differed significantly from chance. In both species the direction of forelimb preferences was strongly sex-related. Since the reaction to novelty as well as stress can potentially modulate laterality (e.g., [88-90]) in our study we used non-experimental method to assess limb preferences: animals were video recorded during their usual activity and their unimanual actions were not artificially evoked. The process of the video recording outside the cages did not lead to the elevated vigilance or stress in animals, since they were habituated to the continual human presence. In this regard we assumed that the effects of novelty or experimental design were kept to minimum. However, as in any study of captive animals, we cannot fully exclude a possibility that animals were stressed in some sort of chronic way owing to captivity, and that this may influenced their manual laterality [90].

### Differences between sexes

We found that grey short-tailed opossums display oppositely directed group–level biases in two sexes, with females preferring to use their left forelimbs in all types of unimanual behaviour and males exhibiting right-forelimb preferences in feeding on living and non-living food, as well as supporting the body in the tripedal stance, but showing only a rightward skew, but not a significant bias in nest-material collecting (Figure 2). In sugar gliders females also showed a greater use of the left paw across all types of manual actions, whereas, males did not display any preference as a group with a slight bias toward right-forelimb use (Figure 4). Revealed sex-related tendencies also reflect the differences in the distribution of individual preferences between sexes (see Figures 1 and 3). The only behaviour where the sex effect on the strength of laterality was shown is nest-material collecting in sugar gliders (females were lateralized stronger than males).

A number of studies on primates also report the effect of the animals’ sex on motor laterality (e.g., [48,50,51,55]). However, in some primate species sex affects only the strength of limb preferences [91,92], and in many others no differences in preferential hand use between males and females was found at all [54-60]. At the same time, non-primate quadrupeds seem to display more pronounced and contrasting sex differences in manual laterality. Similarly to grey short-tailed opossums (present study) domestic cats [20,93], dogs [22,61], Mongolian gerbils [10] and horses [25,27], have been previously reported to show forelimb preferences in opposite directions in the two sexes. In the light of marsupial data it could be suggested that in quadrupeds limb preferences are more susceptible to such a factor as the subjects’ sex, than primarily bipedal species: the red-necked wallabies ― the species with bipedal locomotion as the preferred gait ― shows significant population–level limb preference and we failed to reveal any sex effect either on the direction, or on the strength of manual biases [45], whereas in obligatory quadrupedal marsupials the direction (in grey short-tailed opossums) and even the presence (in sugar gliders) of group–level preference depends on animals’ sex.

In many primates, including humans, the common tendency has been noted: in variety of tasks left-hand preference seems to be more characteristic of males and right-hand preference — of females (e.g., [46,48,50,52,53]). This shift is considered to be a result of a hormonal influence [94]. Of particular interest is the fact that in all non-primate placentals, which males and females as sub-groups show motor preferences in the opposite directions, males also prefer to use their left forelimb and females also tend to employ their right forelimb [10,20,22,26,61]. In quadrupedal marsupials sex differences were distributed in an opposite way: male grey short-tailed opossums showed the right-forelimb preference in most of the observed unimanual behaviours and in male sugar gliders we found no preference with a slight rightward trend, whereas females of both species displayed group-level preferences of the left forelimb. Thus, there seems to be notable differences in how the sex affects manual laterality, especially the direction of preferences, between the placental and marsupial mammals. The potential explanation of these differences is one of the marsupials’ brain peculiarities ― the alternative way of interhemispheric connections in the absence of the corpus callosum [84,95]. In placental mammals the size of the corpus callosum was found to be inversely linked with asymmetry expression [96-98]. Furthermore, the sexual dimorphism in the corpus callosum size seems to be associated with differences in motor laterality in males and females [99-102]. The alternative way of interhemispheric connection may result in a fundamentally distinct mechanism of sex effect on limb preferences in marsupials compared to placentals. Further research is surely needed to investigate the actual nature of sex differences in the manual laterality in marsupials as well as in placentals, because in the latter it is also largely unclear.

Nest-material collecting was the type of behaviour specifically influenced by the animals’ sex. In grey short-tailed opossums it was the only studied behavior, where no group level bias was found in males; females, at the same time, showed significant group–level preference in collecting of the nest material similar to those observed in other types of behaviour. In sugar gliders manual preferences for the nest-material collecting was stronger in females than in males ― this is the only case, when the sex affected the strength of laterality in the present study. Potentially, these differences between the sexes are linked with more involvement of females in the nest-material collecting as compared to males in both species, which may be related with maternal care [103,104]. Indeed, in our study we noted that females of both species were more often and actively engaged in the nest building behaviour. Several studies in humans showed that left-hand cradling bias is characteristic of women while men do not display any significant preference when holding infants [105,106]. Here also, greater involvement of women in infant care as compared to men could be one potential explanation for this phenomenon.

### Effect of subjects’ age

In contrast to sex, age has not been found to affect either the direction or the strength of limb preferences in both quadrupedal marsupials studied. However, our samples included opossums in age from two months old and sugar gliders ― from six months old; thus, limb preferences of subjects at the earliest ages were not explored here. For this reason, we could not entirely exclude the age-related differences in manual laterality of these species. Among placental mammals the age effect on manual laterality appears to be an unstable category across species. In some species, similarly to our results, no interaction of individuals’ age and forelimb preference was found [41,56,58,62-64,107]. A number of reports, however, showed the increase [65-69,93] or decrease [46,70-72] of hand preferences with subjects’ age.

### Effect of task characteristics

The complexity of task was another factor that we tested to influence the forelimb preferences. It was proposed [73] that tasks of high level of complexity (“high-level tasks”, e.g., rapid and/or precise motor actions) are more suitable to reveal population–level biases, than tasks of low level of complexity (“low-level tasks”, e.g., simple reaching). One of the clearest examples of these two types of tasks could be simple reaching of a motionless food item versus live prey catching. For instance, when reaching for a piece of raisin or marshmallow, squirrel monkeys did not display significant population preference. In contrast, when catching a live goldfish the same individuals showed a group–level preference of the left hand [78]. The individual hand preferences were also found to get stronger with increase of task complexity in a number of primate species [53,70,75-77]. In our study, however, both grey short-tailed opossums and sugar gliders demonstrated no differences in the direction or strength of manual preferences between feeding on non-living food (low-level task according to Fagot and Vauclair [73]) and catching live insects (high-level task). It was noted that relative simplicity/complexity of a manual task for a particular species is a speculative category [22,36,85]. However, we assume that for grey short-tailed opossums and sugar gliders feeding on non-living food and catching live insects potentially differed in the relative degree of complexity. In case of feeding on live insects, subjects displayed natural prey-catching behaviour, i.e., searched and grasped freely moving insects. When feeding on non-living food, on the contrary, subjects simply reached stable food item from the bowl. Apparently, prey catching required more rapid and skilled movements and greater involvement of visuo-spatial attention, than simple reaching of food. The absence of task complexity effect in two quadrupedal marsupials is consistent with the hypothesis that non-primate mammals differ from primates in sensitivity to task-related factors [85]. In domestic cats no differences in paw preferences was found between reaching when sitting or standing vs. clinging vertically ― tasks, which was showed to differ significantly in their difficulty for the experimental subjects. Konerding et al. [85] suggested that the sensitivity to postural demand associated with task difficulty did not emerge until after primates diverged from other mammals.

The postural origins theory [108-110] states that primate handedness initially evolved with adaptations for feeding in arboreal species, in which the left hand was preferentially used for visually guided prey capture actions, while the opposite hand took the dominating role in the body support. These two lateralized functions were most necessary for arboreal primates, whereas with evolution of a terrestrial life style postural demands became less important and both hands became involved in different kind of manipulation. Examples of separation of manipulative and supportive functions between forelimbs have been later described for a number of primate species [49,111,112]. However, in either grey short-tailed opossums or sugar gliders we did not found such a limb specialization for food manipulation or supporting the body in the tripedal stance. The direction of group preferences for maintaining of the tripedal stance resembled those for reaching tasks. Grey short-tailed opossums are good climbers, but are largely terrestrial [113,114], while sugar gliders are arboreal marsupials highly adapted for foraging on tree branches [114,115]. Despite of their arboreality, sugar gliders still do not display opposite forelimb preferences for feeding, including live prey catching, and supporting the body when hanging tripedally. It seems, that unlike arboreal primates, in arboreal marsupials manual preferences evolved not as a functional adaptation for unimanual feeding with simultaneous posture support.

### Interspecies comparison: the effect of posture

A comparison of the strength of forelimb preferences between the two quadrupedal species revealed significant differences in feeding on non-living food, although not in the rest types of behaviour. In feeding on non-living food sugar gliders were significantly more strongly lateralized than grey short-tailed opossums. When separated by sex, female sugar gliders were significantly more strongly lateralized than female grey short-tailed opossums in feeding on non-living food and nest-material collecting, but no difference was shown for males.

Many primates showed the increase of manual preferences in bipedal position in comparison to quadrupedal position [8,37,38,42,63,74,116,117]. Furthermore, in prosimians the species with more vertical body orientation tend to be more strongly lateralized in motor behaviours than those of more horizontal postural habits [8,40,118]. It has been argued, that in primates the bipedal postural habit in a species enhances preferred hand use, whereas quadrupedal locomotion tend to hinder the expression of handedness [8,37,42,44]. Marsupials can be used as a model to test whether this hypothesis is applicable for non-primate mammals. In red-necked wallabies we have previously showed that the bipedal posture favours the individual and population motor preferences within the species [45]. Basing on primate bipedalism hypothesis, the influence of species typical posture on the expression of limb preferences in various species, i.e., postural effect on interspecific level, in marsupials can be predicted. The comparison of limb preferences between the marsupial species differing in their postural habits gives us some evidence in support of this proposal.

**Figure 5:**
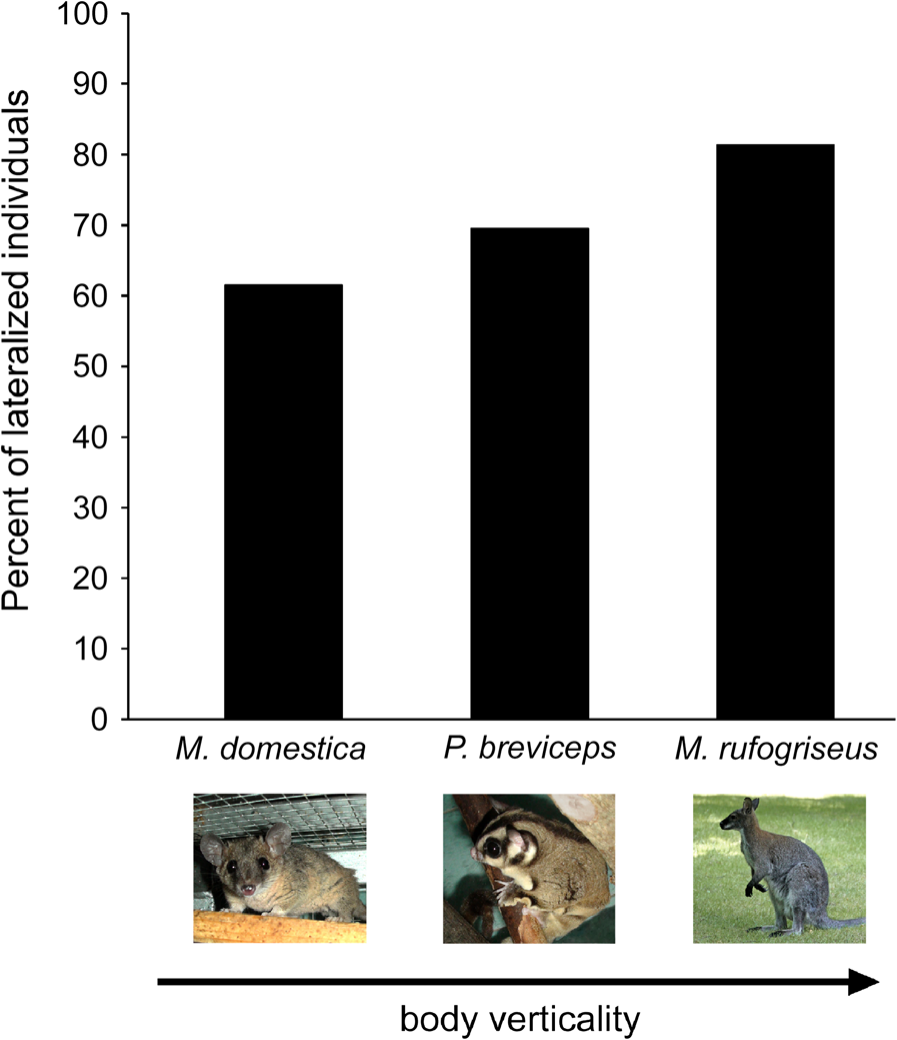
**Percent of lateralized individuals in marsupials with different species-typical body orientation. **The figure contains data on grey short-tailed opossums (*Monodelphis domestica*), N = 26, sugar gliders (*Petaurus breviceps*), N = 23, and red-necked wallabies (*Macropus rufogriseus*), N = 27. Each bar represents the percent of subjects, which showed significant forelimb preferences based on individual z scores (for feeding on non-living food in grey short-tailed opossums and sugar gliders (Table 1, 2) and for feeding on grass/hay from the bipedal position in red-necked wallabies [44]). The percentage of lateralized individuals increases in the row of marsupial species from terrestrial quadruped (grey short-tailed opossum) and arboreal quadruped (sugar glider) to biped (red-necked wallaby), i.e. together with enhancement of body verticality.

The most bipedal species characterized with the most vertical body orientation from the species studied is, apparently, red-necked wallaby, which use bipedal locomotion as the preferred gait. Two obligatory quadrupeds studied here have slightly distinct postural characteristics. Grey short-tailed opossums are largely terrestrial [113,114] with usually horizontal their body’s long axis orientation. Arboreal sugar gliders, in contrary, often climb vertical surfaces and are typically observed with relatively more vertical body orientation [114,115]. Our observations showed that when feeding with use of the both forelimbs sugar gliders often took a bipedal position, whereas in grey short-tailed opossums it was observed very rarely. The type of behaviour comparable across these three species seems to be feeding on non-living food, in which laterality was explored in all three species. For wallabies, feeding from the bipedal position was chosen, because our results indicated that in this species feeding from quadrupedal position is not a representative type of the unimanual actions in terms of limb preferences [45]. It can be traced that the percentage of lateralized individuals decreases from red-necked wallabies (81%) to sugar gliders (70%) and grey short-tailed opossums (62%). This decline coincides with the decrease of relative verticality of body orientation and the degree of bipedality across species (Figure 5). In addition, the mean ABS–HI, reflecting the strength of manual preferences independently of the bias direction, also decreases in the row of studied species with less vertical body orientation from red-necked wallabies (ABS–HI = 0.53) to sugar gliders (ABS–HI = 0.49) and to grey short-tailed opossums (ABS–HI = 0.34). Thus, the proportion of lateralized individuals and the strength of manual laterality in marsupials tend to be enhanced with more upright species-typical posture.

In primates, the degree of bipedality of a species was proposed to correlate with manifestation of population directional bias in hand use [37,39,41,42]. Somewhat similar tendency could be traced in marsupials. The red-necked wallabies with bipedal locomotion as the preferred gait showed significant population-level preferences for actions performed from the bipedal position [45]. Quadrupeds ― brush-tailed possums [81], grey short-tailed opossums and sugar gliders (present study), on the contrary, did not display unilateral population-level preferences. According to McGrew and Marchant’s classification [119] bipedal red-necked wallabies do show task specialization since the majority of subjects prefer to use the same limb in a certain task, whereas quadrupedal brush-tailed possums, grey short-tailed opossums and sugar gliders do not.

All together our results indicate that the effect of posture on the manual laterality is not a unique characteristic of primate taxon, but could also take place in non-primate mammals such as marsupials. Further investigation of limb preferences in marsupials, especially in species with bipedal locomotion, is required, however, before generalized conclusions regarding postural effect on the particular ways of evolution of motor laterality in this taxon can be drawn. To the best of our knowledge, to date only four marsupial species have been studied in the aspect of manual preferences: grey short-tailed opossum (Didelphimorphia) ([82], present study), brush-tailed possum [81], red-necked wallaby [45] and sugar glider (present study) (Diprotodontia). These reports indicate that marsupials are characterized by a diversity of laterality patterns among the species: in grey short-tailed opossums and sugar gliders the direction of forelimb preferences is strongly sex-related (but has species differences), brush-tailed possums have no population-level motor preference (the effect of sex was not tested), while red-necked-wallabies have. Even less we know about sensory lateralization in marsupials; here the only studied species are stripe-faced dunnart (Dasyuromorphia) exhibiting left-eye preference for response to a fear-stimulus [79], and hairy-nosed wombat demonstrating right-side skew in reaction to the sound [80]. Both of these biases were considered to be consistent with results of studies in placentals and other vertebrates. Existing data on motor asymmetry also indicate a number of similarities between marsupial and placental mammals, e.g., common factors influencing laterality manifestation like sex and posture of a subject as well as species-typical postural habit.

## Conclusions

We showed that in two marsupial quadrupeds the direction of forelimb preferences is strongly sex-related: male grey short-tailed opossums showed right-forelimb preference in most of the observed unimanual behaviours and no group preference, though with a slight rightward trend, was found in male sugar gliders; whereas females in both species displayed a group-level preference of the left forelimb. Such a distribution of preferences between two sexes is not typical for placental mammals in which left-hand preference is usually more characteristic of males and right-hand preference ― of females. One potential explanation of the difference in sex effect on laterality between the placentals and marsupials is the alternative way of interhemispheric connections in the marsupial brain.

No influence of subjects’ age on limb preferences as well as no separation of manipulative and supportive functions between the forelimbs was found. We failed to reveal any differences between the two tasks of potentially differing complexity, which supports the hypothesis non-primate mammals to differ from primates in their sensitivity to task-related factors.

Interspecies comparison demonstrated that species typical posture could be the factor determining different manual laterality manifestation in marsupials (Figure 5). The vertical body orientation and the bipedality tend to favour the expression of individual and population forelimb preferences in marsupials at both intra- and interspecific levels. Our findings showed for the first time that the effect of posture on the manual laterality is not a unique characteristic of primate taxon, but could also take place in non-primate, and even non-eutherian mammals such as marsupials.

## Methods

### Species

The grey short-tailed opossums are South American didelphid marsupials that are solitary, omnivorous, and nocturnal [114], and reach the reproductive maturity at about 4–5 months [120]. This quadruped is usually observed on the ground and is not highly adapted for arboreal life; however, it is able to climb the trees well [113,114]. Their food in the wild mainly consists of small rodents, insects, carrion, seeds, and fruits. The sugar gliders are social, omnivorous and largely nocturnal animals characterized by a quadrupedal locomotion and inhabiting Australia, New Guinea and some nearby islands [114,115]. They reach reproductive maturity at about 8–15 months in males and at about 12 months in females [121]. Besides plant products such as sap, nectar and pollen, the natural diet of this arboreal species includes insects, arachnids, and small vertebrates. The nest building behaviour is typical for both *M. domestica* and *P. breviceps* and they both display a similar behaviour pattern during nest-material collecting: passing the material via the forefeet to the hind feet and then to the tail [103,114,122].

### Subjects

A total of 26 grey short-tailed opossums (14 females, 12 males) ranging in age from two months to three years and three months were studied. All animals were captive born and were housed solitary in Moscow Zoo, Russia under a 15 h/9 h light/dark period. Animals were fed daily at the beginning of dark phase; the basis of the ration was chopped raw beef, boiled eggs, and porridge.

We studied 23 sugar gliders (12 males, 11 females) aged between five months and five and a half years, housed in the same zoo. The subjects were captive born and were kept in mixed-sex social groups ranging from three to nine individuals. Sugar gliders were housed under a 12 h/12 h light/dark period and fed daily a diet mainly consisting of assorted chopped fruits with yogurt at the beginning of dark phase.

In addition, animals of both species were provided daily with fresh nest-material (hay and thin paper strips) and fed with live insects (crickets, *Gryllus sp*., tenebrionid beetle larvae, *Zophobas morio*). Insects were placed on the organic litter on the cages floor where they moved freely. So, the marsupials were allowed to search and catch the prey like in a natural foraging situation. The non-living food, in contrast, was placed in bowls.

Individual identification of sugar gliders (opossums were housed solitary) was based on artificial marks on the animals’ bodies. Before the data collecting was started, zoo staff individually marked each subject by cutting off a small area of the hair on different parts of the body, mainly on the tail. No markings were made on the subjects’ forelimbs or the frontal part of the body to avoid the influence of marking on laterality of forelimb use. Additionally, the most of the sugar gliders were already familiar with the procedure of marking, because they have been previously marked in a similar way for other behavioural studies. Therefore, animals were not under much stress during and after this procedure. Despite of this we waited two days after the marking of animals and before the starting of data collecting; hence, we assumed that the possible influence of marking was kept to the minimum. Marking and video recording proceeded with permission from administration of Moscow Zoo, and did not lead to any changes in the usual housing conditions.

### Procedure

The data for this study were collected in March 2009, 5–6 h per day during 14 consecutive days for each species. We video recorded all types of animals unimanual behaviour. However, unimanual behaviours, which were very rarely observed, were not included in the analysis. Unimanual behaviours were not artificially evoked and the animals were video recorded during their usual activity. Since the both studied species are nocturnal, all video recordings were carried out during the dark phase of light cycle, when the animals were active. The video cameras Sony DCR-SR-220E and Sony DCR-HC-17E in the NightShot mode with infrared lighting were used for this aims. To minimize the researcher’s influence on the behaviour of animals, video recording was conducted outside the cages through the glass wall of the cage. Two people carried out the video recording simultaneously, but of different subjects and in random order in different days of the data collecting. In total each individual was observed for 7 – 9 h depending on its activity.

Since different types of unimanual actions could be more or less suitable to assess forelimb preferences (e.g., [49,73,123]), the investigation of only one task may be not enough to characterize manual laterality of a species [36,124]. We explored all types of unimanual behaviours which we observed usually in the chosen marsupial species in captive conditions. From video recordings we scored the number of times a subject used the right or the left forelimb in four distinct types of behaviours per species. The manual behaviours studied in grey short-tailed opossums and sugar gliders were similar, however they differed very slightly between two species.

For both species we assessed preference in unimanual food reaching, separately in feeding on non-living food and catching live insects. In case of opossums chopped meat served as non-living food, since other components of the diet such as egg and porridge they fed without any limb-use. In sugar gliders chopped fruits served as non-living food. Reaching act was counted when the subject maintained all limbs on the substrate prior to using one of the forelimbs to grasp the food item.

We further evaluated asymmetrical forelimb use in supporting the body in the tripedal stance. The tripedal stance in a subject was scored when both hindlimbs and one of the forelimbs was in contact with the substrate, while the other forelimb was held in the air. The forelimb used by a subject for body support in the tripedal stance was registered. It should be noted, that the animals of the two species took such a stance in different ways. In the tripedal position the grey short-tailed opossums stand on hind-limbs and one of the forelimbs on the ground with the other forelimb in the air. This posture is very similar to those previously described for Mongolian gerbils [10]. Both the grey short-tailed opossums and the Mongolian gerbils took this position by raising one forelimb from the initial quadrupedal position. For the sugar gliders the tripedal stance was reached differently: initially hanging upside down on the horizontal branch or cage roof on four limbs, the individual released one of the forelimbs and continued to hang on three other limbs.

Another unimanual action, which we assessed in the grey short-tailed opossums and the sugar gliders, was nest-material collecting. We noted which of the forelimbs was used by the animal to grasp a nest material item (piece of paper or hay). The act was counted only when the subject maintained all limbs on the substrate before grasping. It should be noted that from the total sample size of sugar gliders sufficient data on nest-material collecting were obtained only for 19 individuals (10 females and 9 males), as the rest of animals very rarely or never displayed this type of behaviour.

To obtain discrete responses for each of behaviours in both studied species, a single unimanual act was taken into account if a subject moved into a new location (made at least five steps) after the time when the previous response was scored. Cases when the unimanual action was performed from a biased position, for example when the animal’s body was initially turned to one side, were discarded from the analysis.

### Data analysis

Some authors criticize the use of unequal numbers of unimanual acts per individual in statistical analysis [125]. To maximize comparability of between-individual scores, we obtained an equal number of unimanual acts per individual within each behaviour type before the analysis began. We used only the first *n* acts recorded per individual and for this *n* was taken the minimal number of acts obtained per individual in the sample in the respective type of behaviour. According to a Kolmogorov–Smirnov test, our data were not normally distributed. For this reason, we used nonparametric tests (two-tailed) for all analyses. The following set of statistics for data analysis was used.

First, the degree of individual lateral bias was identified by calculating an individual handedness index (HI) using the formula: (left forelimb use−right forelimb use)/(left forelimb use + right forelimb use). The HI ranges from −1.0 to +1.0, indicating right and left forelimb bias, respectively. Values close to zero indicate equal use of right and left hand. We used the absolute value of each subject’s HI (ABS–HI) to estimate the strength of individual forelimb preference independently of the direction of lateral bias.

Second, in order to evaluate the direction of forelimb preference, a binomial *z* score was calculated in each type of behaviour for each individual based on the total number of left and right forelimb responses. Negative z scores indicated a right preference; positive z scores ― a left preference. Based on their z scores, the subjects were categorized as left-hand preferent (*z* ≥ 1.96), right-hand preferent (*z* ≤ −1.96) or ambipreferent (−1.96 < *z* <1.96) in a given type of behaviour. Chi-square analysis was then performed to determine whether the distribution of left-preferent, right-preferent and ambipreferent individuals differed significantly from expected by chance 25:25:50 distribution (e.g., [14,39,41]).

Third, the influence of such factors as age, sex and type of unimanual behaviour was explored (on the basis of individual HI scores for direction and ABS–HI scores for strength). The associations between age of individuals and both the direction and the strength of forelimb preferences were determined using Spearman rank–order correlation. The effects of sex on both the direction and the strength of forelimb preferences in each type of behaviour were examined with Mann–Whitney *U* tests. Friedman’s test (with post hoc Dunn’s tests for between-pair comparisons) was carried out to estimate the effect of behaviour type on laterality. Manual laterality in feeding on non-living food and feeding on live insects was separately compared using Wilcoxon matched-pairs signed rank test.

One-sample Wilcoxon Signed-rank tests based on the individual HI scores with hypothetical median of zero were performed to explore population–level preference in all four types of behaviour. Consistency in forelimb use across types of behaviour was estimated with Spearman rank-order correlation. Finally, interspecies comparison on the direction (using HI scores) and the strength (using ABS–HI scores) of manual laterality between similar types of behaviour was performed using Mann–Whitney *U* tests. We adopted alpha value at 0.05 for all analyses.

### Ethical statement

This study does not include any study of human subjects or non-human primates, thus does not need any specific adherence to the Declaration of Helsinki or Weatherall report. As for the work with other subjects, this work, which only implies pure observations on animals, did not require any permission according to local rules and laws in Russia.

## Competing interests

The authors declare that they have no competing interests.

## Authors’ contributions

GA and KK designed the study, collected and analysed primary data, wrote the manuscript. YM designed and organized the study, discussed data and wrote the manuscript. All authors read and approved the final manuscript.
